# Gender differences in outpatients with dementia from a large psychiatric hospital in China

**DOI:** 10.1186/s12888-022-03852-z

**Published:** 2022-03-21

**Authors:** Jiaojiao Zhou, Chengwei Guo, Li Ren, Dandi Zhu, Wenfeng Zhen, Saina Zhang, Qing’e Zhang

**Affiliations:** The National Clinical Research Center for Mental Disorders & Beijing Key Laboratory of Mental Disorders, Beijing Anding Hospital & the Advanced Innovation Center for Human Brain Protection, Capital Medical University, No. 5 Ankang Hutong, Xicheng District, Beijing, 100035 China

**Keywords:** Dementia, Outpatient, Gender, Age, Number of visits, Comorbidity

## Abstract

**Background:**

The sociodemographic characteristics and clinical features of dementia patients in psychiatric hospitals have not been thoroughly studied in China. This study aimed to explore the psychiatric outpatient attendance of dementia patients at a psychiatric hospital in China, with particular emphasis on gender differences.

**Methods:**

This retrospective study examined outpatients with dementia from January 2013 to August 2019 using data in the Observational Medical Outcomes Partnership Common Data Model (OMOP-CDM) in Beijing Anding Hospital. Age, sex, number of visits, use of drugs and comorbid conditions were extracted from medical records.

**Results:**

Nine thousand four patients were recruited from a specific outpatient clinic of a hospital in Beijing, and the mean number of visits was 6.92. There were 3,433 (38.13%) male patients and 5,571 (61.87%) female patients. The most common comorbidities were generalized anxiety disorder, nonorganic insomnia, delusional disorder and depressive disorder. The proportion of patients using antidementia was the highest, with the rate of 68.3%, followed by benzodiazepines (48.83%), antipsychotics (45.43%), antidepressants (22.24%) and nonbenzodiazepines (19.96%). Patients with dementia showed a significant gender difference in average age (t = 6.36, *P* < 0.0001). Compared to male patients, female patients had a higher number of visits (7.40 ± 12.90 vs 6.15 ± 10.50, t = 4.81, *P* < 0.0001). There were significant differences in comorbidity composition between male and female patients (t = 23.09, *P* < 0.0001).

**Conclusions:**

Our present findings suggested significant gender differences in the proportion of age, number of visits and comorbidity composition in outpatients with dementia.

## Introduction

Dementia is a clinical syndrome characterized by progressive impairment in cognitive and functional abilities [1]. There are 9.9 million new cases of dementia every year, and it is projected that in 2050, more than 115 million people will experience dementia worldwide [2, 3]. With the ageing population increasing worldwide, the prevalence of dementia among elderly individuals is also on the rise. A cross-sectional epidemiological study showed that the prevalence of dementia was 5.60% [95% confidence interval (CI) 3.50–7.60] in 2019 for individuals aged 65 years or older [4]. However, the clinical situation of dementia patients attending a psychiatric outpatient department has not been thoroughly studied in China yet.

It is well known that dementia has a negative impact on the quality of life of patients and poses a significant financial burden on their family and society [5]. The estimated global cost of dementia in 2015 was 818 billion dollars, which had increased by 35% since 2010, and these costs are estimated to increase to approximately 2 trillion dollars by 2030 [6]. Meanwhile, the calculated annual costs of dementia in China increased from 0.9 billion dollars in 1990 to 47.2 billion dollars in 2010 and are projected to reach 69.0 billion dollars by 2020 and 114.2 billion dollars by 2030 [7]. Besides, dementia is a major cause of morbidity and mortality among elderly people [8]. Unfortunately, dementia is incurable, and the existing pharmacological and nonpharmacological interventions can only delay its progression [9, 10]. The number of dementia patients in China accounts for about 25% of all patients with dementia worldwide [11], resulting in a huge challenge for family members, policy makers and health-care professionals [12]. Thus, accurate forecasts and estimates of dementia prevalence will aid policy makers, health care and families providers in planning for the economic, social, and health burden of this formidable and costly disease.

Patients with dementia often experience behavioural and psychological symptoms (BPSDs), including depressive symptoms, anxiety, apathy, sleep problems, irritability, psychosis, wandering, elation and agitation. Sex could affect the risk of dementia [13, 14]. A systematic review of 75 observational studies showed that the prevalence of dementia in 2013 was higher in women than in men, with a ratio of 1.65:1 [15]. It has been reported that men express more aggressive and regressive behaviours than women, while women are more likely to suffer from depressive symptoms [16]. Current guidelines recommend that nonpharmacological interventions should be the first choice to reduce BPSD, even though their clinical efficacy has been questioned [17]; psychotropic drug administration is generally used as a second-line treatment [18]. In recent years, patients with dementia have tended to visit psychiatric clinics to treat BPSD due to the heavy burden on caregivers of individuals with dementia [19]. There is evidence of high levels of psychotropic drug use among older people with dementia in Finland [20]. Nevertheless, low rates of follow-up visits are commonly observed in patients with dementia. However, psychiatric comorbidity and psychotropic medication in psychiatric outpatients with dementia, especially the gender difference, remains unclear in China.

Thus, we conducted this study to investigate the sociodemographic characteristics and clinical features (e.g., the number of visits, drug treatments and comorbid conditions) in patients with dementia and examined the gender differences, which may provide useful information for the prevention and treatment of dementia, popularization of science, support of patients’ family members, and formulation of intervention measures information.

## Methods

### Setting and design

This retrospective, cross-sectional study was conducted from January 2013 to August 2019. The present study included 9,004 patients aged 45 or older who visited the clinic in the Department of Psychiatry at Beijing Anding Hospital. The patients were diagnosed with dementia and comorbidities (generalized anxiety disorder, nonorganic insomnia, delusional disorder or depressive disorder) based on the criteria of the fifth edition of the Diagnostic and Statistical Manual of Mental Disorders (DSM-5). Patients with multiple missing data or important missing data due to incomplete information or invalid outpatient records were excluded. A complete medical history and physical examination were obtained from the patients.

### Statistical analysis

This study was based on data platform in Beijing anding hospital affiliated to the capital university of medical sciencesis, which was a mental health alliance of the beijing-tianjin-hebei region. The Observational Medical Outcomes Partnership Common Data Model (OMOP CDM), developed by the Observational Health Healthcare Data Science and Informatics Alliance (OHDSI: https://ohdsi.org/), was used to create a database based on clinical diagnosis and treatment data. Statistical Package for the R version 4.0.3. was used for all statistical analysis. The level of significance was *P* ≤ 0.05. χ^2^ and t-test analyses were conducted to compare the possible gender differences.

## Results

### Demographic and clinical data

The demographic and clinical data were summarized in Table 1. A total of 9,004 patients with dementia aged ≥ 45 years (mean age, 76.20 ± 10.27 years) were enrolled in our study, including 5571 (61.87%) females and 3433 (38.13%) males.

**Table 1:** Demographic and clinical data of patients with dementia

Variables	Total
Gender	
Female, n (%)	5571 (61.87%)
Male, n (%)	3433 (38.13%)
Age, Mean ± S.D., year	76.20 ± 10.27
Visits	
Average number of visits, Mean ± S.D	6.92 ± 12.06
Number of only one visit	3558 (39.52%)
Number of multiple visits	5446 (60.48%)
Cost per case(US$)	
Medical costs	849.36
Non-medical costs	84.70
Comorbidities	
Generalized anxiety disorder, n (%)	4337 (48.17%)
Nonorganic insomnia, n (%)	3604 (40.03%)
Delusional disorder, n (%)	2618 (29.08%)
Mental disorder, n (%)	2377 (26.40%)
Major depressive disorder, n (%)	2292 (25.46%)
Prescribed drugs	
Antidementia drugs, n (%)	6150 (68.30%)
Antipsychotics, n (%)	4118 (45.43%)
Antidepressants, n (%)	2003 (22.24%)
Benzodiazepines, n (%)	4397 (48.83%)
Non-benzodiazepines, n (%)	1797 (19.96%)
Mood stabilizers, n (%)	689 (7.65%)

The average number of outpatient visits was 6.92, with an SD of 12.06. These findings indicated that 60.48% of all patients had at least two outpatient visits, and 39.52% had only one visit. The mean cost per case for outpatients was approximately US$ 934.06, consisting of US$ 849.36 for direct medical costs and US$ 84.70 for direct nonmedical costs.

The most common comorbidities were generalized anxiety disorder (48.17%), nonorganic insomnia (40.03%), delusional disorder (29.08%) and depressive disorder (25.46%). Among all prescribed drugs, the proportion of drug use was the highest in antidementia (68.30%), followed by benzodiazepines (48.83%), antipsychotics (45.43%), antidepressants (22.24%), and nonbenzodiazepines (19.96%). The smallest proportion (7.65%) had treatment with mood stabilizers.

### Gender differences in patients with dementia

The gender differences in average age, average number of visits and comorbidities in patients with dementia were shown in Table 2.

**Table 2:** Gender differences in average age, average number of visits of patients and comorbidities with dementia

Variables	Female	male	t	P
Age, Mean ± S.D	76.73 ± 9.88	75.32 ± 10.82	6.36	< 0.0001
Average number of visits, Mean ± S.D	7.40 ± 12.90	6.15 ± 10.50	4.81	< 0.0001
Comorbidities			23.09	< 0.0001
Generalized anxiety disorder	2760 (49.5%)	1541 (46.1%)		
Nonorganic insomnia	2271 (40.8%)	1306 (38.0%)		
Delusional disorder	1674 (30.0%)	910 (26.5%)		
Mental disorder	1451 (26.0%)	903 (26.3%)		
Major depressive disorder	1537(27.6%)	719 (20.9%)		

The average age of the patients was 75.32 ± 10.82 years for males and 76.73 ± 9.88 years for females. This study found a significant gender difference in average age (t = 6.36, *p* < 0.0001). In addition, compared to male patients, female patients had a higher number of visits (7.40 ± 12.90 vs 6.15 ± 10.50, t = 4.81, *p* < 0.0001). The top four comorbidities in female patients were generalized anxiety disorder (2,760, 49.5%), nonorganic insomnia (2,271, 40.8%), delusional disorder (1,674, 30.0%), and major depressive disorder (1,537, 27.6%). The top four comorbidities in male patients were generalized anxiety disorder (1,541, 46.1%), nonorganic insomnia (1,306, 38.0%), delusional disorder (910, 26.5%) and major depressive disorder (719, 20.9%). There was a significant difference in comorbidity composition between male and female patients (t = 23.09, *p* < 0.0001).

## Discussion

To the best of our knowledge, this was the first study to investigate the demographic and clinical characteristics of patients with dementia in a psychiatric hospital in China. Our results demonstrated a higher ratio (61.87%) of female dementia than male dementia patients (38.13%), and the mean age of females was significantly higher than that of males, which is consistent with previous studies [21, 22]. The probable reason for this result is women’s longer lifespans [23], as the prevalence of dementia increases with age.

In addition, our data revealed that the cost per case in psychiatric outpatients is low compared to the findings in other studies in China [7]. On the basis of current knowledge, it is difficult to give reasonable explanations for this phenomenon. One possible reason for the difference is that patients with dementia were from a psychiatric hospital rather than a general hospital. Another explanatory factor is that the cost of informal care for patients with dementia was not taken into account. Further studies are needed to interpret the difference costs per cases between the psychiatric outpatients of the present study and that of patients with dementia of a general hospital in China.

The current study also showed that 39.52% of patients with dementia had only one visit, indicating poor outpatient adherence. Patients with dementia usually visit neurologists instead of psychiatrists in China because of concerns about stigma. Dementia is incurable, and the existing pharmacological and nonpharmacological interventions can only delay its progression [9, 10]. Low rates of follow-up visits are commonly observed in patients with dementia. Thus, it is necessary to improve dementia patients' understanding of dementia and increase their outpatient compliance to improve the therapeutic effect of this disease. In addition, gender differences were found in visit frequency. The discrepancies between genders were probably attributed to the higher number of female patients, owing to the greater caution of men in seeking medical attention.

The Initial symptoms of some dementia patients are BPSDs and directly see a doctor in the psychiatric clinic. Some of them are referred to a psychiatric clinic by a neurologist or general practitioner. So, not all of them have been seen by a neurologist, and the diagnosis of dementia can be done by other medical specialists. A further finding of our present study, similar to previous studies [24, 25], was that BPSD was common: generalized anxiety disorder (48.17%), nonorganic insomnia (40.03%), delusional disorder (29.08%) and depressive disorder (25.46%). Meanwhile, our finding that female patients had a significantly higher risk of depression symptoms than male patients was in line with previous studies that have shown that women are more prone to depressive symptoms [16, 26]. Evidently, BPSD are an integral part of dementia. However, apathy and agitation were not included in this study because the outpatient department did not record them, which may have influenced the results. These symptoms could affect the patient's mental behaviour and place a serious burden on the caregiver, which is also the major reason that the patients visit the psychiatric department.

More interestingly, a previous study, involved 1,037 patients (769 female patients) with a median age of 89 years, revealed that the prevalence of prescribed medication classifications was as follows: antidepressant (37.4%), benzodiazepines (39.0%), antipsychotic (22.2%) and antidementia (18.3%) [27]. In contrast, we found the following prevalence of drug use among patients with dementia: antidementia (68.30%), benzodiazepines (48.83%), antipsychotic (45.43%) and antidepressants (22.24%). One possible reason for the difference is the smaller sample size in the previous study and the exclusion of some patients with severe dementia. Besides, the significant differences in age (mean age: 89.0 years vs 76.2 years) and gender composition (female: 74% vs 61.87%) of the participants may also account for the different results of the two studies. Despite the limited evidence, this high utilization rate of psychotropic drugs is most likely an attempt to treat neuropsychiatric symptoms of dementia [28].

The limitations of this study should be noted. First of all, this was a retrospective study over 6 years (2013–2019), and data were collected by sample outpatient medical records. Some important factors, such as course of illness and degree, were not available for assessment. Meanwhile, using of antipsychotics is contrasting with the frequency of psychotic symptoms. However, some diagnoses (e.g., agitation) that may lead to antipsychotic medication have not been assessed. Secondly, since the participants were recruited from a single regional hospital, the findings cannot be generalized to all hospitals. The last but not the least, no further detailed analysis was carried out on the symptoms and medication in psychiatric departments. Future multi-center large sample studies or related epidemiological investigations should be conducted to obtain more data and use more statistical methods to further explore gender differences and the relevant possible influencing factors.

## Conclusions

Our present findings suggested significant gender differences in the proportion of age, number of visits and comorbidity composition in outpatients with dementia. The findings from this study may be helpful to make a thorough evaluation for patients with dementia to identify the influencing factors. Furthermore, follow-up evaluation should be performed after the treatment so that the therapeutic regimens can be adjusted based on each patient’s condition.

## Data Availability

The data used in this study are available from the corresponding author upon reasonable request.

## Abbreviations

OMOP-CDM: Observational Medical Outcomes Partnership Common Data Model
CI: Confidence interval
BPSD: Behavioural and psychological symptom
DSM-5: The fifth edition of the Diagnostic and Statistical Manual of Mental Disorders
